# Singing as training modality within pulmonary rehabilitation for COPD patients may enhance diaphragmatic function: a pilot RCT exploring impact on diaphragmatic mobility and thickness

**DOI:** 10.3389/fphys.2026.1728597

**Published:** 2026-03-04

**Authors:** Daozheng Lv, Zhengtong Qiao, Jiazhen Zhang, Jiayu Xin, Kai Liu

**Affiliations:** 1 School of Special Education and Rehabilitation, Binzhou Medical University, Yantai, China; 2 Rehabilitation Therapy Center, Luoyang Orthopedic Hospital of Henan Province, Luoyang, China; 3 Department of Rehabilitation Medicine, Qingdao Central Hospital, Qingdao, China; 4 College Of Health- Preservation and Wellness, Dalian Medical University, Dalian, China; 5 Department of Rehabilitation Medicine, Qingdao Hospital, University of Health and Rehabilitation Sciences (Qingdao Municipal Hospital), Qingdao, China

**Keywords:** chronic obstructive pulmonary disease, diaphragmatic function, diaphragmatic ultrasound, pulmonary rehabilitation, respiratory muscle training, singing training

## Abstract

**Introduction:**

Previous studies have suggested that singing training (ST) has been proposed as an engaging adjunct to pulmonary rehabilitation (PR) patients with COPD and may improve diaphragmatic function and alleviate symptoms. We aimed to explore whether ST (a structured, facilitator-led group singing-based intervention for COPD) was associated with improvements in diaphragmatic function and related clinical outcomes—including pulmonary function, exercise capacity, health-related quality of life, and psychological symptoms. We hypothesized that ST would improve diaphragmatic function and yield better patient-reported outcomes than health education (HE).

**Methods:**

In this randomized controlled trial (No. ChiCTR2100052874), we conducted with 40 stable COPD patients randomly assigned to either the ST group (n = 20) or the HE group (n = 20). Primary outcomes were ultrasound-derived diaphragmatic function (dynamic and static diaphragmatic excursion and diaphragmatic thickening fraction). Secondary outcomes included respiratory muscle strength (MIP/MEP), pulmonary function (FEV_1_/FVC, FEV_1_predict), exercise capacity (6-min walk distance, 6MWD), health-related quality of life (St George’s Respiratory Questionnaire, SGRQ), and psychological symptoms (Hospital Anxiety and Depression Scale, HADS). We report between-group differences in change (95% CIs) and MCID attainment for 6MWD (≥30 m increase), CAT (≥2-point decrease), SGRQ (≥4-point decrease), and HADS (≥1.5-point decrease); for other outcomes, a pragmatic ≥10% improvement threshold was applied.

**Results:**

Thirty-three patients completed follow-up (ST n = 18; HE n = 15). Compared with HE, ST produced greater improvement in dynamic diaphragmatic mobility (mean SD change 0.7 ± 0.6 vs. 0.1 ± 0.5 cm; difference 0.6 cm, 95% CI 0.1–1.3; *P* = 0.03) and diaphragmatic thickening fraction (DTF; 23.4% ± 39.8% vs. 1.4% ± 35.7%; difference 22.0%, 95% CI −3.4–47.3; *P* = 0.06). For key secondary outcomes, ST showed greater improvements in 6MWD (52 ± 49 vs. 5 ± 46 m; difference 47 m, 95% CI 18–76; *P* < 0.01), SGRQ (−12.6 ± 11.0 vs. −1.8 ± 9.2; difference −10.8, 95% CI −16.8 to −4.8; *P* < 0.01), and HADS-D (−1.6 ± 1.7 vs. +0.7 ± 2.1; difference −2.3, 95% CI −3.5 to −1.0; *P* < 0.01), whereas no between-group differences were observed for FEV1/FVC or FEV1%pred (both *P* > 0.30). Applying a pragmatic ≥10% improvement threshold for diaphragmatic ultrasound outcomes, clinically relevant improvement was achieved by 9/18 (50.0%) vs. 1/15 (6.7%) for dynamic mobility and 13/18 (72.2%) vs. 6/15 (40.0%) for DTF (ST vs. HE).

**Conclusion:**

In this pilot trial, ST was associated with improved diaphragmatic mobility and several clinically relevant outcomes compared with HE. These findings suggest that ST may serve as a relevant adjunct to pulmonary rehabilitation, however larger trials are needed to confirm efficacy.

**Clinical Trial Registration:**

https://www.chictr.org.cn/showproj.html?proj=135209.

## 1 Introduction

Chronic Obstructive Pulmonary Disease (COPD) is a prevalent chronic respiratory disease characterized by progressive, irreversible airflow limitation (Christenson et al., 2022). Pulmonary rehabilitation (PR), with core components of exercise training and disease-specific education, is recommended for symptomatic COPD and improves exercise capacity, symptoms, and health-related quality of life (Patel, 2024). However, PR remains markedly underused worldwide due to barriers to referral, access, uptake, and completion, and because some patients report low motivation or willingness to engage in conventional physical exercise training. (Lahham and Holland, 2021; Chung et al., 2025; Holland et al., 2021). The lack of motivation is particularly prominent in traditional physical exercise training, especially when some patients experience low mood or lack interest in physical activity (Oates et al., 2019). These gaps have driven interest in feasible and engaging adjuncts or alternative training modalities that can be delivered in community settings while targeting breathing control and respiratory muscle function.

The diaphragm is the primary inspiratory muscle (Fogarty, Mantilla, and Sieck, 2018). However, in patients with COPD, its initial contraction is less efficient due to lung hyperinflation, forcing it to operate at unfavorable length-tension relationships (Decramer, 1997). This results in decreased diaphragmatic contraction and fatigue, a condition referred to as “diaphragmatic dysfunction” (Wei et al., 2022). Singing training (ST) in this study refers to a structured, facilitator-led group singing-based intervention for COPD, incorporating physical warm-up, breathing and vocal exercises, graded song practice, and relaxation/mindfulness (Lord et al., 2010; Kaasgaard et al., 2022a). Existing studies on ST has been evaluated as an adjunct to, or an alternative component within, pulmonary rehabilitation programs, but an optimal approach has not yet been defined, nor has ST been established as a standard training modality within PR (Fang et al., 2022).

To date, evidence from heterogeneous and largely small-sized randomized trials and systematic reviews suggests that ST may improve selected patient-reported outcomes and aspects of physical health in COPD (McNamara et al., 2017; Fang et al., 2022; Philip et al., 2024). While several studies have employed measures such as MIP and MEP to explore diaphragmatic function in ST for COPD, the evidence remains limited and inconsistent (Silva et al., 2024; Torres-Castro et al., 2025; Kaasgaard et al., 2022b). These findings suggest that singing may engage respiratory muscles, but further research is needed to elucidate the specific impact on diaphragmatic mobility and its clinical implications. Therefore, we conducted a 12-week pilot randomized controlled trial to assess the effects of ST on ultrasound-derived diaphragmatic function and on pulmonary function, exercise capacity, symptoms, mood, and health-related quality of life.

## 2 Materials and methods

### 2.1 Study design and setting

**FIGURE 1:**
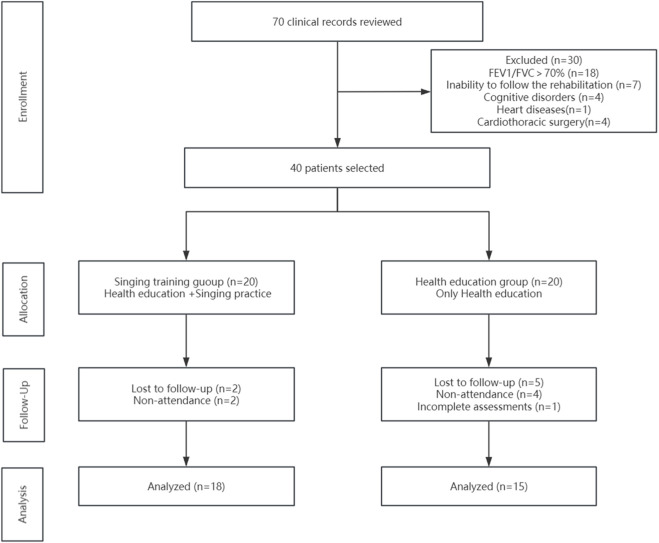
Abbreviations: Flowchart showing participant’s progress through the study.

This prospective, single-center, parallel-group randomized controlled pilot study was conducted in Qingdao, China, between March 2023 and September 2023. The study was performed at the Department of Rehabilitation Medicine, Qingdao Municipal Hospital, a tertiary public hospital affiliated with the University of Health and Rehabilitation Sciences. The study followed CONSORT guidelines for randomized trials. The study flow diagram is presented in Figure 1, which shows the recruitment, allocation, and follow-up processes for participants in this trial.

### 2.2 Protocol

This study was reviewed and approved by the Ethics Committee of Qingdao Municipal Hospital (Approval No. 2023 Pro-review No. Y020), and clinical trial registration was completed (Registration No. ChiCTR2100052874).

### 2.3 Recruitment

40 stable COPD patients were randomly assigned to either the ST group (n = 20) or the Health Education (HE) group (n = 20), with “HE” referring to structured disease education sessions provided by trained healthcare professionals. Participants were recruited from six communities within Qingdao, China. The recruitment process involved conducting community outreach presentations and posting recruitment flyers at local healthcare centers and public areas within each community. These efforts aimed to raise awareness of the study and inform potential participants about the research.

Eligible participants were then screened based on the inclusion and exclusion criteria for stable COPD patients. Those who met the criteria were invited for further screening, which included a comprehensive assessment to confirm their suitability for participation. Informed consent was obtained from all participants before enrollment.

The demographic and baseline clinical characteristics of both groups are detailed in Table 1. There were no significant differences between the groups in terms of age, sex, BMI, or other baseline characteristics (*P* > 0.05).

**TABLE 1:** Study participants.

Basic characteristics	Singing training (n = 18)	Health education (n = 15)	*P*-value
n	18	15	-
Age (year)	65.7 ± 7.6	66.4 ± 4.7	0.75
Male sex	15 (83.4%)	10 (66.7%)	-
Height (m)	1.7 ± 0.1	1.7 ± 0.1	0.90
Weight (kg)	72.44 ± 14.47	76.90 ± 11.00	0.34
GOLD classification			
Class 1	0 (0%)	0 (0%)	-
Class 2	12 (66.7%)	7 (46.6%)	-
Class 3	5 (27.8%)	7 (46.6%)	-
Class 4	1 (5.6%)	1 (6.7%)	-
BMI (kg·m^-2^)	24.8 ± 3.6	26.4 ± 3.8	0.23
Smoking history (year)	28.8 ± 18.0	27.7 ± 16.7	0.15
Medical history (year)	10.2 ± 9.8	10.0 ± 7.2	0.95
FEV_1_/FVC	44 ± 19.1	50.3 ± 10.5	0.26
FEV_1_%predict	60.3 ± 20.0	51.2 ± 18.9	0.19
Comorbidities, n (%)			
Cardiovascular disease	8 (44.4%)	6 (40.0%)	-
Hypertension	9 (50.0%)	8 (53.3%)	-
Diabetes mellitus	6 (33.3%)	4 (26.7%)	-
Medication			
Inhaled long-acting muscarinic antagonists	12 (66.7%)	8 (53.3%)	-
Inhaled long-acting β2-agonists	11 (61.6%)	9 (60%)	-
Inhaled Corticosteroids	3 (16.7%)	1 (6.7%)	-

Data are presented as median (IQR) or n (%) unless otherwise stated; BMI, body mass index; GOLD, Global Initiative for Chronic Obstructive Lung Disease FEV_1_%pred, forced expiratory volume in one second; FVC, Forced Vital Capacity FEV_1_%pred, forced expiratory volume in one second % predicted; Medical history (years) refers to the duration (in years) that participants have had a history of COPD., cardiovascular disease includes coronary artery disease, heart failure, and arrhythmias.

### 2.4 Randomization process

Randomization was conducted by an independent researcher using sequentially numbered, opaque, sealed envelopes. Allocation concealment was maintained until interventions began. The investigator who performed randomization was not involved in assessments or data analysis. Participants and facilitators were aware of group allocation, while outcome assessors and data analysts were blinded to group assignment.

### 2.5 Eligibility criteria

Inclusion criteria were: 1) age <80 years; 2) diagnosis according to GOLD criteria, with FEV_1_/FVC <70% after inhalation of bronchodilators for inclusion in primary screening; 3) negative bronchodilator test to exclude asthma; 4) stable condition with no acute exacerbations in the 4 weeks prior to enrollment; 5) ability to undergo subgroup randomization; and 6) ability to participate in regular aerobic exercise and complete the 6-min walking test.

Exclusion criteria were: 1) unstable cardiovascular disease, such as recent hospitalization for decompensated heart failure, unstable angina, or myocardial infarction within the past 6 months; 2) vocal disorders (e.g., vocal cord nodules, chronic laryngitis); 3) severe cognitive impairment; 4) participation in aerobic training (or other disease-related systematic physical training) within the past 6 months; 5) severe hypoxic respiratory failure that could not be safely managed during pulmonary rehabilitation (e.g., resting SpO_2_ < 88% on room air or requiring long-term supplemental oxygen therapy); 6) insufficient education and comprehension levels to qualify for ST (Qiao et al., 2024).

### 2.6 Detail of interventions

Both groups received the same structured COPD HE delivered weekly by a trained respiratory nurse, in addition to usual care. The ST group additionally attended facilitator-led ST sessions, whereas the control group received HE only. Thus, the comparison was HE + ST vs. HE alone. Participants in the ST group received a 12-week intervention, which involved two 60-min sessions per week. Based on the practices outlined in McNamara et al. (2017), which included training durations ranging from 60 min, 2 times per week, to 90 min, 1–2 times per week, we selected a 60-min session, delivered twice a week, in line with local standard practices. This duration allows for a balanced integration of singing training, breathing control exercises, and relaxation, while minimizing potential physical strain on participants (McNamara et al., 2017). Sessions were conducted by facilitators specialized in respiratory therapy and music-based interventions.

Detailed ST:

Part I: Preparation and Warm-up (about 20 min).

1. Breathing Awareness and Positive Thinking Exercise (5 min):

Instruct the patient to sit in a comfortable position with one hand on the abdomen and one hand on the chest. Guide them to close their eyes and feel their natural breathing, then consciously perform slow and deep inhalations and exhalations with the goal of feeling the forceful sensations of inhalation and exhalation and focusing their mental attention on their body.

2. The warm-up process consists of 10 min of respiratory muscle:

Sternocleidomastoid, obliques, intercostals stretching, and 5 min of combined chest and abdominal breathing exercises. This was followed by gentle vocal warm-ups such as lip trills (lip blowing), humming, and sustained exhalation of “sssss” and “shhhh” to activate the respiratory muscles and vocal organs.

Part II: Core ST (approximately 35 min).

1. Breathing control exercises

Slow inhalation-long exhalation exercises: Instruct the patient to inhale deeply through the nose for 3 s, and then exhale through the mouth by slowly retracting the lips, prolonging the exhalation time to 6–10 s as much as possible.

2. Music Theory Learning Stage

Tone name C D E F G A B and the ascending and descending notes of each note, singing name 1 2 3 4 5 6 7 and the ascending and descending notes of each note, so as to facilitate the patient’s recognition of music.

3. Song singing (30 min)

Choose songs with soothing melodies, steady rhythms, and moderate phrase lengths. Initially choose old songs and folk songs with short phrases, and gradually introduce songs with slightly longer phrases as the patient’s ability improves.

Part III: Relaxation and organization (about 5 min).

Practice slow, deep breathing exercises while singing an extremely soothing song or engaging in group humming to gradually restore heart rate and breathing rate to resting levels. Use the Brog scale to assess the exertion level of this training session. Adaptations were made for COPD patients to avoid vocal strain, excessive respiratory load, or breath-holding.

For transparency and reproducibility, we also provide a more detailed description in the Supplementary Appendix.

- A soothing song and relaxation were used to reduce physical tension after prolonged breath control and vocalization.
- The cool-down supported gradual return of breathing pattern toward resting levels and helped participants transition from exercise to rest.
- The final song also provided low-intensity practice to consolidate breathing/vocal strategies learned earlier in the session.

#### 2.6.1 Detailed HE

The HE group attended 12 weekly 60-min sessions focusing on disease education, medication adherence, and lifestyle modification, without breathing or physical exercises.

### 2.7 Assessments

Assessments were performed at both the beginning and the end of the 12-week rehabilitation program. Three domains were evaluated: clinical characteristics, diaphragmatic function, and functional performance indicators.

### 2.8 Outcomes

Results include clinical characteristics, primary outcomes, and secondary outcomes.

Primary outcome: Change in diaphragmatic function (mobility and thickening fraction) measured by ultrasound during quiet and deep breathing.

Secondary outcomes: Respiratory muscle strength; Pulmonary function (FEV_1_, FVC, FEV_1_/FVC); Exercise capacity (6-Minute Walk Distance, 6MWD); Health status (COPD Assessment Test, CAT; St George’s Respiratory Questionnaire, SGRQ); Psychological symptoms (Hospital Anxiety and Depression Scale, HADS).

We report between-group differences in change from baseline to post-intervention with 95% CIs and, for selected outcomes with established Minimal clinically important difference (MCID), the proportion achieving prespecified MCID thresholds (defined as the smallest change perceived as important and sufficient to justify a change in management). For outcomes without well-defined MCIDs, we additionally applied a transparent, pragmatic ≥10% improvement-from-baseline threshold, following prior *post hoc* analyses by Kaasgaard et al., as an exploratory indicator of potentially clinically relevant change.

#### 2.8.1 Clinical characteristics

Baseline demographic and clinical data were collected, including age, sex, smoking status, smoking history, medication use, and comorbidities. Comorbidities recorded included hypertension, diabetes mellitus, and coronary artery disease. Anthropometric data (height, weight, and BMI) were also measured.

#### 2.8.2 Primary outcome

DM/DTF

Diaphragm ultrasonography was performed using a Mindray M9 color Doppler ultrasound system (Mindray, Shenzhen, China). Participants were examined supine with the head of the bed elevated 0°–30°. Diaphragmatic thickening fraction (DTF) was measured at the zone of apposition using a 5–12 MHz linear probe in B-mode (end-inspiration and end-expiration). Diaphragm mobility (DM) was measured using a 1–5 MHz convex probe in M-mode. DM and DTF were evaluated by ultrasound during quiet and deep breathing. DM was calculated as the vertical displacement of the diaphragm between end-expiration and end-inspiration (Yakut et al., 2023).

$$DTF=\frac{\left({Thickness}_{Deep\;inspiration}-{Thickness}_{static\;expiration}\right)}{{Thickness}_{static\;expiration}}\times 100\%$$

#### 2.8.3 Secondary outcome

##### 2.8.3.1 MIP/MEP

Maximum inspiratory pressure (MIP) and maximum expiratory pressure (MEP) were measured with a spirometer mouthpiece pressure acquisition system to evaluate respiratory muscle strength. MIP and MEP are outcome measures used to assess inspiratory and expiratory muscle performance. They represent integrated indicators of the structural and functional status of the respiratory muscles, rather than being merely simple pressure measurements (Neves et al., 2014; McCool et al., 1997). Participants exhaled to residual volume, followed by maximal inhalation or forced exhalation, and maintained pressure for 2–3 s (Fayssoil et al., 2022). MIP/MEP was assessed using a BH-AX-MAPG portable integrated pulmonary function system.

##### 2.8.3.2 Pulmonary function tests

Pulmonary function tests spirometry was performed following ATS/ERS guidelines using CHESTGRAPH HI-101 (OMRON, Japan). Parameters included FVC, FEV_1_, FEV_1_/FVC ratio, and FEV_1_% predicted. Measurements were taken 20 min after inhalation of 400 μg salbutamol (Georgopoulou et al., 2022).

##### 2.8.3.3 6MWD

Exercise capacity was assessed using the 6-min walk distance (6MWD); an increase indicates improvement, and the prespecified MCID was a ≥30-m increase (Polkey et al., 2013; Zeng et al., 2018; Holland and Nici, 2013).

##### 2.8.3.4 CAT

The COPD Assessment Test (CAT; range 0–40) was used to assess health status, where higher scores indicate worse symptoms/health status. A decrease indicates improvement, and the prespecified MCID was a ≥2-point decrease (Jones et al., 2009).

##### 2.8.3.5 SGRQ

The St George’s Respiratory Questionnaire (SGRQ; range 0–100) assessed disease-specific health-related quality of life, where higher scores indicate worse health status (Jones et al., 1992). A decrease indicates improvement, and the prespecified MCID was a ≥4-point decrease (Jones et al., 2009; Jones, 2005).

##### 2.8.3.6 HADS

The Hospital Anxiety and Depression Scale (HADS) assessed anxiety (HADS-A) and depression (HADS-D) symptoms; higher scores indicate greater symptom burden (Zigmond and Snaith, 1983). A decrease indicates improvement, and the prespecified MCID for each subscale was defined as a ≥1.5-point decrease (Wynne et al., 2020; Smid et al., 2017; Puhan et al., 2008)

### 2.9 Sample size and statistical analysis

This study used 40 participants to assess feasibility and estimate variance for future trials. Assuming a within-group effect size of 0.8 for diaphragmatic mobility, 80% power, α = 0.05, and 15% dropout, a sample of 20 per group was considered adequate.

All analyses were performed using SPSS 26.0 and GraphPad Prism 10.1.2. Missing data were handled using last observation carried forward (LOCF). Continuous variables are presented as mean ± SD. Between-group comparisons used independent t-tests; within-group pre–post comparisons used paired t-tests. A p-value <0.05 was considered statistically significant.

## 3 Results

### 3.1 Participant characteristics

Twenty patients were included in each of the ST group and the HE group. Two participants withdrew from the ST group during the intervention period (2/20, 10%), leaving 18 participants who completed the trial (90%). Five participants withdrew from the health education group due to personal reasons (5/20, 25%; four withdrew on their own and one withdrew for planned lung decongestive surgery), leaving 15 participants who completed the trial (75%). Overall, 33/40 participants completed the study (82.5%). A comparison of the baseline data showed no statistically significant differences in baseline characteristics between the two groups (*P* > 0.05).

### 3.2 Primary outcome: diaphragmatic function

#### 3.2.1 Diaphragmatic mobility (DM)

At baseline, deep-breathing diaphragmatic excursion was ∼5 cm bilaterally and above published healthy-adult lower limits of norma for deep-breathing excursion (Boussuges et al., 2021). Because hyperinflation in COPD can alter diaphragmatic mechanics and excursion during deep inspiration relative to healthy reference cohorts (Cao et al., 2022), we focused on within-person change. In the absence of established MCIDs for diaphragm ultrasound outcomes, we additionally report the proportion of participants achieving a pragmatic ≥10% improvement-from-baseline threshold as an exploratory responder indicator.

Between groups, improvement in dynamic diaphragmatic mobility was greater in ST than HE (between-group difference in change +0.6 cm, 95% CI 0.1 to 1.3; *P* = 0.03; Table 2). Within groups, dynamic DM increased from 5.0 ± 0.8 cm to 5.7 ± 0.6 cm in ST (*P* < 0.01) and remained unchanged in HE (5.4 ± 1.1 to 5.4 ± 1.0 cm; *P* = 0.88). Using the ≥10% threshold, 9/18 (50.0%) in ST versus 1/15 (6.7%) in HE achieved improvement.

**TABLE 2:** Effect of singing training (ST) on primary and secondary outcomes in COPD patients: Comparison with healthy education (HE).

Variable	ST group (N = 18)				HE group (N = 15)				ST vs. HE		
	Baseline	Post	P value	MCID/MID achieved	Baseline	Post	P value	MCID/MID achieved	Groups-change	95% CI	MCID/MID P value
Primary outcomes											
Static diaphragmatic mobility (cm)	2.2 ± 0.7	2.4 ± 0.7	0.89	5/18 (27.7%)	2.4 ± 0.7	2.5 ± 0.5	0.64	2/15 (13.3%)	+0.3	−0.3 to 0.7	0.28
Dynamic diaphragmatic mobility (cm)	5.0 ± 0.8	5.7 ± 0.6	<0.01	9/18 (50.0%)	5.4 ± 1.1	5.4 ± 1.0	0.88	1/15 (6.7%)	+0.6	0.1 to 1.3	<0.01
Diaphragmatic thickening fraction (DTF, %)	60.3 ± 24.3	83.7 ± 38.8	0.02	13/18 (72.2%)	46.0 ± 28.1	47.4 ± 33.1	0.86	6/15 (40.0%)	+22.0	−3.4 to 47.3	0.07
Secondary outcomes											
Maximum inspiratory pressure (MIP, mmHg)	57.9 ± 14.9	66.8 ± 15.5	0.01	5/18 (27.8%)	59.1 ± 17.3	57.2 ± 15.0	0.32	2/15 (13.3%)	+10.8	6.3 to 15.2	0.28
Maximum expiratory pressure (MEP, mmHg)	67.5 ± 19.7	73.1 ± 16.8	0.01	6/18 (33.3%)	74.1 ± 18.0	70.7 ± 15.2	0.08	1/15 (6.7%)	+9.2	4.1 to 14.3	0.07
FEV1/FVC	53.4 ± 12.9	57.1 ± 15.0	<0.01	10/18 (55.6%)	47.2 ± 11.6	48.1 ± 11.3	0.52	4/15 (26.7%)	2.8	−2.2 to 7.8	0.09
FEV1%pred (%)	67.9 ± 16.4	63.8 ± 16.2	0.34	2/18 (11.1%)	53.2 ± 18.2	50.0 ± 18.0	0.29	1/15 (6.7%)	−0.9	−5.9 to 4.2	0.57
6-min walk test (6MWT, m)	421 ± 45	473 ± 73	0.01	10/18 (55.6%)	407 ± 48	402 ± 48	0.66	1/15 (6.7%)	+47	18 to 76	<0.01
COPD assessment test (CAT, points)	19.7 ± 6.2	11.7 ± 4.2	0.01	15/18 (83.3%)	18.2 ± 8.3	17.5 ± 8.3	0.43	3/15 (20.0%)	−7.3	−10.3 to−4.3	<0.01
St George’s respiratory questionnaire (SGRQ)											
SGRQ-total score	35.1 ± 10.8	22.5 ± 10.9	0.01	15/18 (83.3%)	28.7 ± 12.4	26.9 ± 12.0	0.21	5/15 (33.3%)	−10.8	−16.8 to−4.8	0.01
SGRQ-symptoms score	45.3 ± 18.9	27.1 ± 13.3	0.01	13/18 (72.2%)	41.3 ± 19.2	41.7 ± 16.6	0.86	4/15 (26.7%)	−18.6	−30.5 to −6.8	0.01
SGRQ-activity score	48.6 ± 13.9	36.3 ± 15.6	0.01	14/18 (77.8%)	43.1 ± 12.5	38.7 ± 15.9	0.86	6/15 (40.0%)	−8.0	−18.0 to −1.3	0.03
SGRQ-impact score	35.1 ± 10.8	22.5 ± 10.9	0.01	11/18 (61.1%)	28.7 ± 12.4	26.9 ± 12.0	0.21	4/15 (26.7%)	−9.7	−16.7 to −2.7	0.05
Hospital anxiety and depression scale											
Anxiety (HADS-A)	5.3 ± 3.1	3.0 ± 2.4	0.01	13/18 (72.2%)	4.9 ± 3.0	4.6 ± 3.0	0.26	1/15 (6.7%)	−2.0	−3.0 to −1.1	<0.01
Depression (HADS-D)	4.7 ± 2.3	3.2 ± 1.5	0.01	9/18 (50.0%)	4.5 ± 2.6	5.3 ± 2.1	0.14	2/15 (13.3%)	−2.3	−3.5 to −1.0	0.03

Values are presented as mean ± SD, unless otherwise specified. Within-group changes and between-group differences in change scores (post-intervention minus baseline) were analyzed using appropriate parametric tests, with 95% confidence intervals (CI) reported. MCID, achieved is presented as the number of participants achieving the minimal clinically important difference divided by the total number of participants (%).The 6-min walk test (6MWT) is reported in meters without decimals.ST, singing training; HE, healthy education; DTF, diaphragmatic thickening fraction; MIP, maximum inspiratory pressure; MEP, maximum expiratory pressure; CAT, COPD, assessment test; SGRQ, St George’s Respiratory Questionnaire; HADS, hospital anxiety and depression scale.

Static diaphragmatic mobility changed minimally and the between-group difference in change was not significant (95% CI −0.3 to 0.7; *P* = 0.32; Table 2). Within groups, static DM changed from 2.2 ± 0.7 to 2.4 ± 0.7 cm in ST (*P* = 0.89) and from 2.4 ± 0.7 to 2.5 ± 0.5 cm in HE (*P* = 0.64). Using the ≥10% threshold, 5/18 (27.8%) in ST and 2/15 (13.3%) in HE achieved improvement (Table 2).

#### 3.2.2 Diaphragmatic thickening fraction (DTF)

At baseline, the diaphragmatic thickening fraction (DTF) was well above the commonly used threshold for impaired thickening (DTF <20%) (Boon et al., 2013).

Because no validated MCIDs have been established for diaphragm ultrasound outcomes in COPD, we additionally report the proportion of participants achieving a pragmatic ≥10% improvement-from-baseline in DTF as an exploratory responder indicator.

**FIGURE 2:**
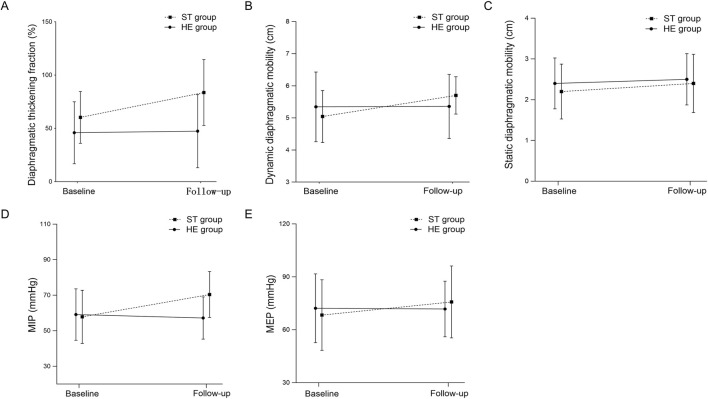
Effects of singing training on diaphragmatic function and respiratory muscle strength Abbreviations: Changes in diaphragmatic thickening fraction (DTF, %) **(A)**, dynamic diaphragmatic mobility **(B)**, static diaphragmatic mobility **(C)**, maximum inspiratory pressure (MIP) **(D)**, and maximum expiratory pressure (MEP) **(E)** from baseline to follow-up in the singing training group (STG) and the health education group (HEG). Data are presented as mean ± SD. STG, singing training group; HEG, health education group.

Between groups, the change in DTF tended to be larger in ST than HE but did not reach conventional statistical significance (between-group difference in change +22.0%, 95% CI −3.4 to 47.3; *P* = 0.06; Table 2). Within groups, DTF increased in ST (60.3% ± 24.3% to 83.7% ± 38.9%; *P* = 0.02) with no meaningful change in HE (46.0% ± 28.1% to 47.4% ± 33.1%; *P* = 0.86). Using the ≥10% threshold, 13/18 (72.2%) in ST versus 6/15 (40.0%) in HE achieved improvement.) (Table 2). Changes in diaphragm-related indicators over time are shown in Figure 2.

### 3.3 Secondary outcome

#### 3.3.1 Maximum inspiratory pressure (MIP) and maximum expiratory pressure (MEP)

Studies indicate that both MIP and MEP are significantly lower in COPD patients than in healthy individuals (Khalil et al., 2014). There are marked differences in MIP predictive values across different populations (Sachs et al., 2009), and no clear MCID value has been established to date. To aid clinical interpretation, we report responder analyses for MEP using a pragmatic ≥10% increase from baseline for measures. For MIP, we report the proportion achieving a literature-based MID of ≥17 cm H_2_O (≈12.5 mmHg) improvement (Kaasgaard et al., 2022b).

Between groups, MIP improved more in ST than HE (between-group difference in change +10.8 cm H_2_O, 95% CI 6.3 to 15.2; *P* < 0.01; Table 2). Within groups, MIP increased in ST (57.9 ± 14.9 to 66.8 ± 15.5 cmH_2_O; *P* = 0.01) and did not improve in HE (59.1 ± 17.3 to 57.2 ± 15.0 cmH_2_O; *P* = 0.32). Using the ≥10% threshold, 15/18 (83.3%) in ST versus 2/15 (13.3%) in HE achieved improvement. Using the MID of ≥17 cmH_2_O, achieved a clinically meaningful improvement 5/18 (27.8%) in ST versus 2/15 (13.3%) in HE (Table 2).

Between groups, MEP also improved more in ST than HE (between-group difference in change +9.2 cmH_2_O, 95% CI 4.1 to 14.3; *P* < 0.01; Table 2). Within groups, MEP increased in ST (67.5 ± 19.7 to 73.1 ± 16.8 cmH_2_O; *P* = 0.01) with no significant change in HE (74.1 ± 18.0 to 70.7 ± 15.2 cmH_2_O; *P* = 0.08). Using the ≥10% threshold, 6/18 (33.3%) in ST versus 1/15 (6.7%) in HE achieved improvement. Changes in MIP and MEP over time are shown in Figure 2.

#### 3.3.2 Pulmonary function test

At baseline, both groups demonstrated airflow obstruction, with mean FEV1/FVC values well below the commonly used diagnostic threshold of 0.70 (Table 2). Baseline FEV1% predicted was ∼53–57%, consistent with moderate (GOLD 2) airflow limitation (50%–79% predicted). We therefore report between-group differences in change and, because no validated MCIDs are established for changes in FEV1/FVC or FEV1% predicted, exploratory responder analyses using a pragmatic ≥10% relative improvement-from-baseline threshold for both indices (Donohue, 2005; Mortensen et al., 2026).

Between groups, the difference in change was not statistically significant (between-group difference in change +2.8%, 95% CI −2.2 to 7.8; *P* = 0.31; Table 2). Within groups, FEV1/FVC increased in ST (53.4% ± 12.9% to 57.1% ± 15.0%; *P* < 0.01) and changed minimally in HE (47.2% ± 11.6% to 48.1% ± 11.3%; *P* = 0.52). Despite this improvement, post-intervention mean values remained consistent with airflow obstruction. Using the ≥10% threshold, 10/18 (55.6%) in ST versus 4/15 (26.7%) in HE achieved improvement.

Between groups, no significant difference in change was observed (between-group difference in change −0.9%, 95% CI −5.9 to 4.2; *P* = 0.72; Table 2). Within groups, FEV1% predicted decreased in ST (67.9% ± 16.4% to 63.8% ± 16.2%) and in HE (53.2% ± 18.2% to 50.0% ± 18.0%) (Table 2). Using the ≥10% threshold, 2/18 (11.1%) in ST versus 1/15 (6.7%) in HE achieved improvement.

#### 3.3.3 6-Minute walk test

Between groups, 6MWT distance improved more in the ST group than in the HE group (between-group difference in change 47 m, 95% CI 18 to 76; *P* < 0.01) (Table 2). In the ST group, distance increased from 421 ± 45 m at baseline to 473 ± 73 m post-intervention (*P* = 0.01), whereas it changed little in the HE group (407 ± 48 m to 402 ± 48 m; *P* = 0.66). Using a minimal clinically important difference (MCID) of 30 m, 10/18 (55.6%) in the ST group and 1/15 (6.7%) in the HE group achieved a clinically meaningful improvement in walking distance (Table 2).

#### 3.3.4 CAT

Between groups, the reduction in CAT score favored the ST group. The CAT score decreased by approximately −8 points in the ST group and by −0.7 points in the HE group, yieldi ng a between-group difference in change of −7.3 points (95% CI −10.3 to −4.3; *P* < 0.01) (Table 2).

**FIGURE 3:**
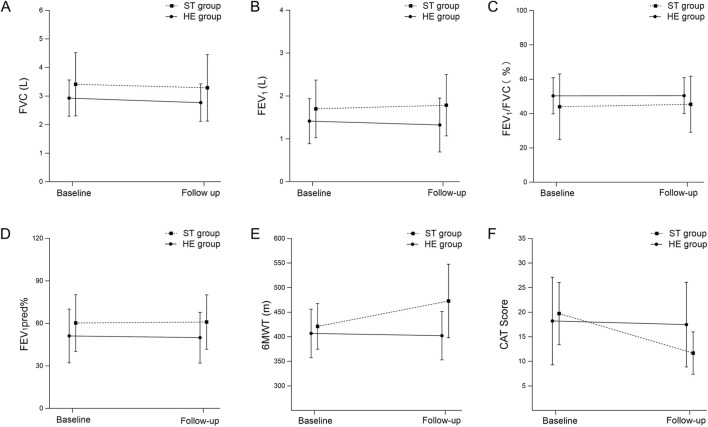
Effects of singing training on pulmonary function, exercise capacity, and symptom burden Abbreviations: Changes from baseline to follow-up in forced vital capacity (FVC) **(A)**, forced expiratory volume in 1 s (FEV_1_) **(B)**, FEV_1_/FVC ratio **(C)**, percent predicted FEV_1_ (FEV_1_%pred) **(D)**, 6-min walk test distance (6MWT) **(E)**, and COPD Assessment Test (CAT) score **(F)** in the singing training group (STG) and the health education group (HEG). Data are presented as mean ± SD. STG, singing training group; HEG, health education group.

Within groups, CAT scores improved significantly in the ST group (from 19.7 ± 6.2 to 11.7 ± 4.2; *P* < 0.01), whereas only a small and non-significant reduction was seen in the HE group (from 18.2 ± 8.3 to 17.5 ± 8.3; *P* = 0.44) (Table 2). With an MCID of two points, 15/18 patients (83.3%) in the ST group and 3/15 (20.0%) in the HE group achieved a clinically meaningful reduction in CAT score. Changes in Pulmonary function test ,6-Minute walk test and CAT over time are shown in Figure 3.

#### 3.3.5 St. George’s Respiratory Questionnaire (SGRQ)

Between the groups, the improvement in the total SGRQ score was significantly greater in the ST group compared to the HE group. In the ST group, the total score decreased from 35.1 ± 10.8 at baseline to 22.5 ± 10.9 post-intervention (P < 0.01), whereas in the HE group, the total score decreased from 28.7 ± 12.4 at baseline to 26.9 ± 12.2 post-intervention (*P* = 0.21). The between-group difference in change was −7.3 (95% CI −10.3 to −4.3; *P* < 0.01) (Table 2).

Using the MCID threshold of four points, 15/18 (83.3%) participants in the ST group and 5/15 (33.3%) participants in the HE group achieved a clinically meaningful reduction in their total SGRQ score (Table 2).

#### 3.3.6 HADS

Between groups, both anxiety (HADS-A) and depression (HADS-D) improved significantly more in the ST group than in the HE group. For HADS-A, the mean change from baseline to post-intervention was −2.3 points in the ST group and −0.3 points in the HE group, resulting in a between-group difference in change of −2.0 points (95% CI −3.0 to −1.1; *P* < 0.01) (Table 2). For HADS-D, mean changes were −1.6 points vs. +0.7 points, with a between-group difference in change of −2.3 points (95% CI −3.5 to −1.0; *P* < 0.01) (Table 2).

Within groups, HADS-A scores decreased significantly in the ST group from 5.3 ± 3.1 to 3.0 ± 2.4 (*P* < 0.01), whereas they remained essentially unchanged in the HE group 4.9 ± 3.0 to 4.6 ± 2.8 (*P* = 0.26). HADS-D scores also improved in the ST group from 4.7 ± 2.3 to 3.2 ± 1.5 (*P* < 0.01), whereas no significant improvement was observed in the HE group.

**FIGURE 4:**
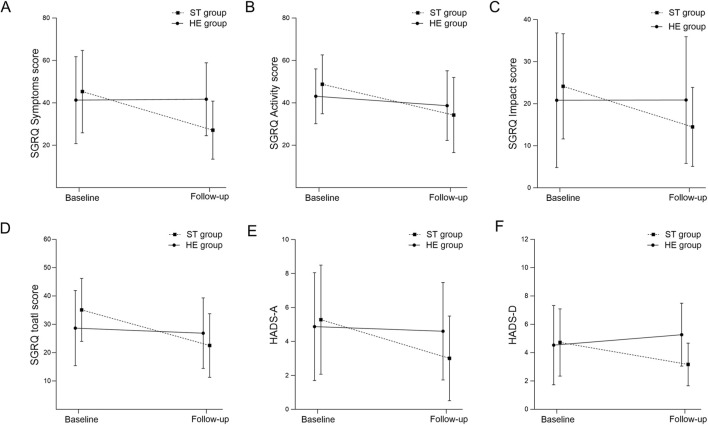
Changes in health-related quality of life and psychological outcomes following singing training Abbreviations: Changes from baseline to follow-up in St George’s Respiratory Questionnaire (SGRQ) symptoms score **(A)**, SGRQ activity score **(B)**, SGRQ impact score **(C)**, SGRQ total score **(D)**, Hospital Anxiety and Depression Scale–Anxiety (HADS-A) **(E)**, and Hospital Anxiety and Depression Scale–Depression (HADS-D) **(F)** in the singing training group (STG) and the health education group (HEG). Data are presented as mean ± SD. STG, singing training group; HEG, health education group.

Using an MCID of 1.5 points for both subscales, 13/18 patients (72.2%) in the ST group and 1/15 (6.7%) in the HE group achieved a clinically meaningful reduction in HADS-A, while 9/18 patients (50.0%) in the ST group and 2/15 (13.3%) in the HE group achieved a clinically meaningful reduction in HADS-D (Table 2). Changes in SGRQ and HADS over time are shown in Figure 4.

## 4 Discussion

In this randomized controlled pilot trial, we compared a 12-week ST program plus usual care with a HE controls condition in patients with stable COPD. This study utilized ultrasound technology to assess diaphragmatic flexibility, providing objective data on diaphragmatic function. The study suggested that. 12-week ST program significantly improved diaphragmatic mobility and other respiratory outcomes in patients with stable COPD.

### 4.1 Diaphragm function

COPD patients are characterized by decreased diaphragm mobility, which related to a progressive decline in lung function and exercise tolerance (Shiraishi et al., 2024). Given this pathological feature, exploring strategies to optimize respiratory muscle activity patterns has become an important direction in pulmonary rehabilitation research (Dominelli and Molgat-Seon, 2022). Although studies on ST in COPD have shown improvements in various outcomes, such as exercise capacity, spirometry, and global respiratory muscle pressures, as well as symptoms and health-related quality of life, as a result, commonly used endpoints provide limited insight into whether ST significantly impacts diaphragmatic mechanics. (Kim, Yeo, and Kim, 2023; Qiao et al., 2024; Philip et al., 2024; Fang et al., 2022; Pacheco et al., 2014). Against this background, our study used diaphragm ultrasound (diaphragmatic mobility and thickening fraction) to provide a direct, muscle-level assessment of the physiological effects of ST.

Major guidance documents (e.g., GOLD and ATS/ERS statements) define the key outcomes of Pulmonary Rehabilitation as improvements in dyspnoea, exercise capacity, and health-related quality of life—all of which are strongly influenced by diaphragmatic dysfunction (Rochester et al., 2023). Hyperinflation and diaphragm flattening limit tidal-volume expansion, increase neuromechanical dissociation, and lead to exertional dyspnoea and premature exercise termination, further impairing these PR outcomes (O'Donnell et al., 2020). Thus, improved diaphragm mechanics are not merely physiological observations but have clear translational relevance to PR goals. In this pilot study, ST was associated with a clearer between-group improvement in dynamic diaphragmatic excursion and thickening than health education, whereas static mobility changes were smaller.

This improvement aligns with the expectations of previous research on ST, as singing requires precise regulation of deep and prolonged respiratory cycles that provide continuous and rhythmic stimulation to the diaphragm. In COPD, lung hyperinflation flattens and shortens the diaphragm, placing it at a mechanical disadvantage on its length–tension curve and increasing the inspiratory neural drive required to generate a given tidal volume (Zhang et al., 2022).

Previous studies highlighting diaphragmatic mobility as a key functional measure in COPD rehabilitation, and diaphragmatic mobility influences dyspnea and exercise tolerance in patients with COPD (Shiraishi et al., 2020; Shiraishi et al., 2021; Paulin et al., 2007). In conjunction with our findings, the study indicates that ST may enhance diaphragmatic function, which in turn could improve various clinical symptoms in COPD patients, such as dyspnea, exercise capacity, and overall respiratory efficiency.

This study shows that compared to the HE group, the ST group showed more significant improvements in MIP and MEP. Our MIP results are consistent with those of Kaasgaard et al. (2022b), where one of the main findings was that the Singing for Lung Health group, in those who achieved SGRQ-MID (Minimal Important Difference in Quality of Life), showed significant improvements in MIP, whereas the Physical Exercise Training group did not show similar changes (Kaasgaard et al., 2022a). Contacting with the findings of Kaasgaard et al., our results suggest that singing training may improve diaphragmatic function and respiratory muscle strength, which in turn enhances health-related quality of life. This training method offers a highly promising supplementary approach to pulmonary rehabilitation for COPD patients, particularly when traditional physical training methods fail to meet patients’ needs, and holds significant clinical relevance. Moreover, physiological models of breathing control have suggested that singing can optimize diaphragmatic recruitment, improving both diaphragmatic function and overall respiratory muscle coordination, which may explain why ST improves inspiratory pressures and related PR outcomes (Lewis et al., 2021).

### 4.2 Pulmonary function tests

Some studies have shown that ST can increase lung capacity by improving breathing patterns (Bonilha et al., 2009; Lewis et al., 2016). The study showed that the 12-week ST program resulted in a significant improvement in the pulmonary function index FEV1/FVC, with a change of +3.7 (from 53.4 ± 12.9 to 57.1 ± 14.99) in the ST group. In contrast, the HE group showed a minimal change of +0.9 (from 47.2 ± 11.6 to 48.1 ± 11.3). The within-group comparison revealed a statistically significant improvement in FEV1/FVC in the ST group (*p* < 0.05), but no significant change was observed in the HE group (*P* > 0.05). Furthermore, the between-group comparison did not reach statistical significance (*P* > 0.05). Theoretically, singing requires continuous adjustment of breathing and intonation and precise control of airflow, which cannot be separated from the diaphragm’s regulation of intra-abdominal pressure and airflow dynamics. ST encourages the use of diaphragmatic breathing to enhance diaphragm engagement, which improves alveolar ventilation efficiency and reduces dead space ventilation, thereby enhancing overall ventilation efficiency (Lu et al., 2020). The subglottal pressure during singing may be similar to the positive expiratory pressure of lip-contracted breathing, which could create resistance to expiratory airflow and potentially help prevent bronchial collapse in early COPD (Philip et al., 2021). Bonilha’s study (Bonilha et al., 2009)demonstrated a significant improvement in lung function indices (Inspiratory capacity) during singing, but there was no significant difference in post-training measurements. This suggests that there is a transient improvement in hyperinflation during singing in patients with COPD, and may explain why patients with non-significant improvement in static lung function are effective in terms of motor function and quality of life.

### 4.3 Functional indicators

Paulin (Paulin et al., 2007) demonstrated that diaphragmatic mobility was positively correlated with 6MWT scores, suggesting that dyspnea, exercise capacity, and quality of life are interconnected and important indicators in COPD patients. Overall, the synergistic improvement of dyspnea, enhancement of exercise capacity, and improvement in quality of life have become the core goals of COPD rehabilitation therapy (Zhang et al., 2024). In the Cochrane systematic review of conventional pulmonary rehabilitation in stable COPD, the pooled between-group improvement in 6-min walk distance (6MWD) compared with usual care was 44 m (95% CI 33–55 m). Real-world data from the UK National Asthma and COPD Audit Programmed (NACAP) further show that approximately two-thirds to 70% of patients undergoing center-based PR achieve at least the 30 m MCID for the 6MWT. In our trial, the mean change in 6MWD in the ST group was 52 m, and 55.6% of patients (10/18) exceeded the 30 m threshold, indicating a moderate effect that is broadly comparable in magnitude to the average 6MWD gains reported with conventional PR, although the proportion of patients reaching the MCID was somewhat lower than that observed in large PR cohorts.

Specifically, the results of this study showed that the 6MWT scores in the ST group were significantly better than those in the health education group, indicating that ST effectively improved patients’ exercise endurance.

Consistent with Lewis et al. (2021) on the physiological link between singing and respiratory control and pulmonary mechanics (Lewis et al., 2021). ST, which involves coordinated deep inhalation and slow exhalation, improved the breathing pattern in COPD patients, reduced dynamic hyperinflation, thereby improving lung ventilation efficiency and alleviating dyspnea. This aligns with previous research suggesting that ST can coordinate deep inhalation and slow exhalation to improve breathing patterns in COPD patients, reduce dynamic hyperinflation, and enhance lung ventilation efficiency, thereby reducing dyspnea.

The larger improvement in 6MWD observed in our study compared with Kaasgaard et al. (2022a) may reflect several complementary factors related to intervention duration, delivery context, and task individualization (Kaasgaard et al., 2022b). While both programmes were delivered twice weekly, the intervention period in our study was longer (12 weeks vs. 10 weeks in Kaasgaard et al., 2022a), resulting in a greater cumulative exposure to singing-based respiratory training. This extended time course may be particularly relevant for consolidating changes in breathing pattern, respiratory muscle coordination, and tolerance to exertion.In addition, the smaller group size in our study allowed greater flexibility for individual adjustment within the group format. For participants who experienced difficulty keeping up with the full musical or respiratory demands, the facilitator could adapt tasks in real time—for example, assigning simpler phrases within the same song, encouraging humming or softer phonation during more demanding passages, and providing posture or alignment cues to reduce unnecessary accessory muscle loading. Such individualised adjustments may help maintain participation, reduce symptom-related interruption, and support gradual physiological adaptation, particularly in participants with lower baseline capacity.Finally, differences in song selection and task grading may also have contributed. In our intervention, songs were selected and adapted to emphasise moderate tempo, predictable meter (e.g., 3/4 or 4/4), manageable phrase length with planned breath points, and transposition to a comfortable pitch range. Within the same song, phrase-level task grading enabled participants with higher capacity to engage in longer phrases or sections requiring greater breath support, while participants with lower capacity focused on simplified phrases, joining selectively and re-entering at planned breath points. This symptom-guided, graded approach may have facilitated more effective engagement of diaphragmatic mechanics while preserving a cohesive and supportive group environment, potentially contributing to the observed gains in exercise capacity.

### 4.4 Depression indicators

COPD not only severely impairs patients’ ability to perform activities of daily living but also significantly impacts their mental health. Psychological issues, such as anxiety and depression, frequently experienced by patients, further exacerbate disease symptoms and negatively affect quality of life (Li et al., 2025). In the present trial, ST was associated with greater improvements in health-related quality of life and psychological status than HE at the between-group level. From a clinical perspective, established MCID thresholds of four points for SGRQ and 1.5 points for both HADS subscales provide a useful benchmark for interpreting these changes. Using these thresholds, 15 of 18 patients (83.3%) in the ST group and five of 15 (33.3%) in the HE group achieved an MCID-level reduction in SGRQ total score, while 13 of 18 (72.2%) versus one of 15 (6.7%) achieved an MCID-level reduction in HADS-A and nine of 18 (50.0%) versus two of 15 (13.3%) achieved an MCID-level reduction in HADS-D. Taken together, these results indicate that a substantial proportion of patients receiving ST experienced clinically meaningful improvements in both disease-specific quality of life and mood, whereas such improvements were much less common in the HE group.

These findings are broadly in line with, but in some respects more pronounced than, previous studies of singing-based interventions and pulmonary rehabilitation in COPD(Philip et al., 2024; Lewis et al., 2016). Trials of “Singing for Lung Health” and group ST have generally reported modest improvements in SGRQ or other health status instruments and small-to-moderate reductions in anxiety and depressive symptoms (Lewis et al., 2016), with mean changes that often approach or slightly exceed the MCID but with a more limited proportion of participants achieving MCID-level improvements. In contrast, the proportion of patients in our ST group reaching MCID thresholds across SGRQ total and domains and both HADS subscales was relatively high, and the between-group differences versus HE were sizeable. On the other hand, our results fall within the range of effects reported for center-based pulmonary rehabilitation, where SGRQ improvements of approximately 8–15 points and MCID responder rates of 40%–80% have been described, although psychological benefits are more variable. Taken together, these comparisons suggest that ST may achieve quality-of-life and mood benefits of a magnitude comparable to those seen in structured rehabilitation programs in a subset of patients, while offering a different and potentially more acceptable format.

Several studies of inspiratory muscle training (IMT) in COPD have shown clear improvements in inspiratory muscle strength and related physiological outcomes, such as increased MIP and exercise capacity, but their benefits on subjective outcomes such as dyspnea, health-related quality of life and psychological status have been inconsistent. Isolated IMT interventions often fail to produce marked improvements in anxiety or depressive symptoms, likely because these outcomes are influenced by psychosocial factors beyond muscle training alone. These discrepancies with our findings may reflect differences in the underlying mechanisms; while IMT targets respiratory muscle function, ST integrates respiratory muscle engagement with pleasurable activity, emotional regulation and structured social interaction. Singing engages controlled breathing patterns that stimulate respiratory muscles and may reduce hyperinflation, while the group-based, music-oriented format provides social support and reduces isolation, which are important determinants of psychological wellbeing (Shi et al., 2024; Leonardi et al., 2022; Mori and Zatorre, 2024).

### 4.5 Singing intervention program

Traditional pulmonary rehabilitation programs are effective but remain markedly underutilized and difficult to access in many settings, with low participation rates and suboptimal long-term adherence (Chen, Zhang, and Curtis, 2022). There is thus a need to explore alternative or adjunctive rehabilitation strategies that are engaging and acceptable to patients. Incorporating ST into rehabilitation programs may help improve patient participation and adherence, thereby supporting long-term respiratory health (McNaughton et al., 2017). Singing training interventions show significant heterogeneity in content, delivery format (e.g., in-person vs. online), course structure, and program names, with variations across different studies and regions (Kim, Yeo, and Kim, 2023). Some studies even adjust the course content based on participant feedback and local conditions, indicating that singing training programs are greatly influenced by their delivery format, facilitator background, and regional cultural characteristics. Due to differences in study designs, it is difficult to directly compare studies, and there is a significant risk of bias (Lewis et al., 2016). Therefore, exploring an optimal, simplified singing training protocol is particularly important.

In recent years, various formats have emerged, including Singing for Lung Health, community choirs, Sing Your Lungs Out, Sing Strong, and SINFONIA (Kim, Yeo, and Kim, 2023). Compared to other singing programs, Singing for Lung Health has more potential as the best protocol due to its structured design and involvement of more researchers. Singing for Lung Health facilitators must undergo professional training, and the program is well-structured. Kaasgaard’s analysis of the Singing for Lung Health program versus Physical Exercise Training with a sample size of 270 participants (Kaasgaard et al., 2022b) is rare in singing training and provides valuable insights for future research. Our study design also references its structured approach.

We agree with Philip et al. (2024), who emphasized that future research should focus on variables such as content, dosage, and duration of the singing interventions (Philip et al., 2024). As mentioned, singing training programs are flexible and vary according to their format and regional specifics. We believe that future research on singing training programs should focus on universal factors within the programs, such as comparing the effects of different rhythms, tones, volume levels, and sentence lengths on singing training, or examining how different intervention durations and cycles impact outcomes. Researchers should also provide more detailed descriptions of the content of the singing training programs and any adjustments made to the songs to better suit COPD patients.

We suggest that future research should focus on identifying universal factors in singing training programs, such as optimal rhythm, tone, volume, and duration, and explore how these factors can be standardized to improve the effectiveness of interventions for COPD patients.

## 5 Strengths and limitations

The primary strength of this study lies in the use of diaphragmatic ultrasound to assess diaphragmatic function, which is a novel and objective approach in COPD rehabilitation research. By employing ultrasound imaging to measure diaphragmatic mobility and thickening fraction, this study provides a more accurate and direct evaluation of diaphragmatic function, which is often overlooked in traditional lung function tests. This method allows for a deeper understanding of the mechanisms underlying the respiratory benefits of ST, particularly its effects on respiratory muscle performance.

But this study has several limitations. First, the sex distribution in our sample was skewed, with a higher proportion of women in the ST group compared to the HE group, which may limit the generalizability of the findings to predominantly male COPD populations. This imbalance may be due to women being more willing to participate in group singing programs, whereas several eligible male patients declined to join, possibly perceiving singing as less appealing than traditional exercise-based training. Second, the intervention period was relatively short, focusing primarily on the effects of a 12-week program. Third, only patients with stable COPD were included; therefore, our findings cannot be generalized to patients in other phases of the disease (e.g., during acute exacerbations or immediately after hospitalization). Fourth, one limitation of this study is the relatively small sample size, which may limit the generalizability of the findings. For these reasons, the results should be interpreted with caution and confirmed in larger, multicenter studies.

## 6 Conclusion

In this randomized controlled pilot trial, our findings suggest that singing training (ST) may enhance diaphragmatic function, which could lead to improvements in key clinical outcomes such as dyspnea, exercise capacity, and overall quality of life in COPD patients. We found that ST not only improves physiological outcomes but also offers psychological benefits, as evidenced by the high proportions of patients in our ST group achieving clinically meaningful improvements in SGRQ, CAT, and HADS scores. We recommend using diaphragmatic ultrasound combined with MIP/MEP measurements as a tool to further explore the effects of ST on respiratory muscle performance. Given its potential to improve both respiratory function and patient outcomes, ST should be considered as part of a comprehensive COPD rehabilitation program. However, because of the modest sample size, single-center design and specific inclusion criteria, the results should be interpreted with caution and confirmed in larger multicenter studies. Future research should explore the long-term durability of these effects, investigate the underlying mechanisms in more detail, and help to refine and standardize singing-based interventions within increasingly personalized pulmonary rehabilitation models for COPD.

## Data Availability

The datasets presented in this study can be found in online repositories. The names of the repository/repositories and accession number(s) can be found in the article/Supplementary Material.
