# Supplementary material for: Singing as training modality within pulmonary rehabilitation for COPD patients may enhance diaphragmatic function: a pilot RCT exploring impact on diaphragmatic mobility and thickness
Source: Front Physiol. 2026 Mar 4;17:1728597. doi: 10.3389/fphys.2026.1728597 (PMC12995629; doi:10.3389/fphys.2026.1728597)
Supplement: Supplementary file 1 [file DataSheet3.docx]

1. **Facilitator specialization**

Regarding the music instructor's specialization, he was a choir conductor at Qingdao Municipal Hospital and also a respiratory rehabilitation therapist with extensive experience in pulmonary rehabilitation and vocal training. His specialization is in contemporary popular music choral performance, which combines bother aspiratory techniques and vocal exercises tailored for patients with respiratory conditions. His experience leading a choir provided valuable insight into group vocalization techniques, fostering a supportive and engaging environment for participants. This experience was crucial for structuring the sessions to maximize participant engagement, improve adherence, and enhance the therapeutic benefits of the singing training for COPD patient

1. **disease-specific adaptations**
   For the benefit of our patients, we have made adjustments to the singing program.
2. Selection of Simpler Songs:
   To accommodate the respiratory limitations of COPD patients and reduce fatigue, simpler songs were selected. These songs typically had slower tempos and less demanding vocal ranges, making it easier for participants to maintain control over their breathing and vocalization without becoming overly fatigued.
3. Emphasis on Posture Training:
   Posture training was incorporated into the sessions to promote optimal diaphragmatic breathing. Proper posture ensures that the participants' lungs can fully expand and that the respiratory muscles are engaged more efficiently, which is especially important for COPD patients who may have compromised lung function.
4. Focus on Breath Control Exercises**:**
   Breath control exercises were a key part of the intervention. These exercises focused on slow, controlled exhalation, which is essential for managing breathlessness and improving respiratory muscle strength. By teaching COPD patients to control their breath, the exercises help reduce anxiety and improve their ability to sustain physical activity.
5. Song-based Breathing Techniques:
   Song-based activities were tailored to include breathing techniques that target COPD-specific symptoms, such as dyspnea. Participants were encouraged to practice slow and deep breathing while singing, which aids in improving lung function and reducing breathlessness during physical exertion.
6. Adjustments in Song Difficulty:
   As participants progressed in their training, the difficulty of the songs was gradually adjusted to increase the challenge while still staying within the participants' physical capabilities. This progression helped to improve participants' endurance and build their confidence without overwhelming them.
7. **It is recommended to engage in some relaxation after singing. Sing a soothing song:**
8. For the patients**:**
   1. Relaxation and tension relief**:** At the end of the training, singing a soothing song helps the patients relax their bodies and reduce post-exercise tension. Throughout the singing training, patients are engaged in various breathing control, posture, and vocal exercises. Particularly during breathing control and prolonged vocalization, patients may become physically tense, focusing on maintaining control and not noticing fatigue. Singing a soothing song at the end helps the patients release muscle tension and avoid discomfort from overusing their respiratory muscles.
   2. Restoring normal heart rate and breathing frequency**:** COPD patients often experience shortness of breath and increased heart rate after physical activity. Singing training, especially during core exercises, requires greater use of breath and lung capacity, which may exacerbate these symptoms. A soothing song, with its slow tempo and relaxing tone, helps patients’ heart rate and breathing frequency gradually return to resting levels. This unconscious relaxation can help COPD patients transition smoothly from the exercise to rest, avoiding excessive tension or discomfort post-training.
   3. Consolidating respiratory and vocal skills**:** A soothing song also provides an opportunity to consolidate the respiratory and vocal techniques learned earlier in the session. Through gentle vocalization and control, patients continue to apply deep breathing and breath control techniques without overloading their bodies, ensuring these skills become integrated into their daily life.
9. **For the therapists:**
   1. Observing patients’ adaptation to training intensity: The soothing song at the end allows patients to rest and transition into a calm state, enabling us to observe their adaptation to the training intensity. This also provides an opportunity to adjust the difficulty of songs and vocal techniques, ensuring the training intensity is appropriate and reducing the risk of unnecessary strain or injury.

In summary, the inclusion of a soothing song at the end of the training serves multiple purposes, including promoting relaxation, restoring normal physiological functions, reinforcing learned techniques, and providing an opportunity for the therapist to assess patient responses and adapt the training accordingly.

1. **Some details of the singing process**

We have expanded the intervention description to improve transparency and reproducibility. In addition to outlining the core session structure (posture/relaxation → breathing exercises → vocal warm-ups → graded song practice → cool-down), we now describe (i) the song-selection rationale and musical parameters used to support slow, controlled expiration and stable pacing (e.g., moderate tempo, predictable meter such as 3/4 or 4/4, manageable phrase length with planned breath marks, and transposition to a comfortable pitch range), and (ii) the individual tailoring procedures applied within the group format.

Specifically, because the group was small, the facilitator could provide targeted adjustments to individual participants during the same song: participants with higher capacity were prompted to use diaphragmatic breathing and more precise vocal placement/technique, whereas participants with lower capacity were encouraged to focus on simplified sections and follow the melody during more demanding passages, prioritizing comfort and symptom-guided pacing. We also clarify the facilitator’s role in maintaining an engaging, supportive group atmosphere to promote participation and adherence, without implying causality regarding outcomes.In group singing, we applied phrase-level task grading within the same song. Participants with higher capacity were invited to sing more demanding phrases (e.g., longer phrases, higher tessitura, or passages requiring more breath support) or to lead on challenging lines, while participants with lower capacity focused on simplified lines/shorter phrases and were encouraged to “follow along” (e.g., joining on easier sections, humming/soft singing during difficult passages, and re-entering at planned breath points). This approach allowed symptom-guided pacing while maintaining a unified group format。
