# Supplementary material for: Singing as training modality within pulmonary rehabilitation for COPD patients may enhance diaphragmatic function: a pilot RCT exploring impact on diaphragmatic mobility and thickness
Source: Front Physiol. 2026 Mar 4;17:1728597. doi: 10.3389/fphys.2026.1728597 (PMC12995629; doi:10.3389/fphys.2026.1728597)
Supplement: Supplementary file 3 [file DataSheet2.docx]

# Singing Repertoire for COPD Patients

In response to your request, we have updated our singing repertoire for COPD patients. The new repertoire is now included in Supplementary Table 1. These songs are categorized into three levels of difficulty, allowing for a progressive training approach. The songs are structured to gradually challenge the patients' vocal capabilities and respiratory control.

## Song Difficulty Levels

The songs are categorized into three levels of difficulty based on the complexity of the melody, rhythm, and vocal range. These levels are designed to accommodate patients at different stages of rehabilitation and vocal strength.

### Level 1: Beginner Songs

The beginner songs are simple, with short phrases and repetitive patterns. These are ideal for initial training, focusing on basic breath control and voice activation.

Example songs include:
- 'Twinkle, Twinkle, Little Star'
- 'Two Little Tigers'
- 'Jasmine Flower'

### Level 2: Intermediate Songs

Intermediate songs have slightly longer phrases and more varied melodies, offering a greater challenge for vocal endurance and breath control.

Example songs include:
- 'The Moon Represents My Heart'
- 'Sweet as Honey'
- 'Fairy Tale'

### Level 3: Advanced Songs

Advanced songs require more vocal range and breath support. These songs challenge both vocal stamina and control, and are appropriate for patients who have developed sufficient strength in their breathing and vocal abilities.

Example songs include:
- 'You and Me' (from 'Crouching Tiger, Hidden Dragon')
- 'Thousands of Songs'
- 'Crossing the Sea to See You'

## Conditions for Advancing to the Next Level

Patients should progress to the next level of songs when they have achieved a consistent improvement in their vocal endurance, breath control, and overall comfort with their current level. Typically, patients will remain at each level for about **4-6 weeks**, depending on their individual progress and comfort level.

Criteria for advancing to the next level include:
- Consistent ability to sing through the entire song without significant breathlessness or discomfort.
- Improvement in the Borg Rating of Perceived Exertion (RPE) scale, indicating a reduction in perceived exertion during the singing session.
- Positive feedback from the patient about vocal endurance and enjoyment.
- Successful completion of 80% of the song repertoire in the current level.

## Reference for Time Frame and Conditions

The time frame of 3-6 weeks at each level is based on general rehabilitation guidelines and previous studies on singing therapy for respiratory patients. It allows patients adequate time to improve their vocal strength and respiratory control before moving to more complex songs.
