# Supplementary material for: Singing as training modality within pulmonary rehabilitation for COPD patients may enhance diaphragmatic function: a pilot RCT exploring impact on diaphragmatic mobility and thickness
Source: Front Physiol. 2026 Mar 4;17:1728597. doi: 10.3389/fphys.2026.1728597 (PMC12995629; doi:10.3389/fphys.2026.1728597)
Supplement: Supplementary file 4 [file DataSheet1.docx]

Detailed Test Methods

1) Lung Function Test

Preparation of the spirometer: Use the spirometer (Breathe Home BH-AX-MAPG Portable All-in-One Lung Function Monitor) to perform the pulmonary function test. At least one environmental calibration and volumetric calibration of the spirometer should be completed every day before the pulmonary function test. Environmental calibration includes measurement of environmental parameters including temperature, humidity, altitude and atmospheric pressure; volumetric calibration is performed using a matching calibration cylinder (3 L).

Patient preparation: Before the examination, patients were instructed to avoid drinking strong tea, coffee, and carbonated beverages, to refrain from eating for 2 h before the examination, and to refrain from strenuous activities for 30 min before the examination. The examinee should avoid wearing clothes with strong binding. Before the examination, the patient's basic information was understood in detail to determine whether he/she was eligible for the pulmonary function examination and to exclude risk factors. Enter the patient's basic information including number, name, gender, date of birth, height and weight, and select the appropriate estimated value according to the region.

Bronchodilator use: albuterol sulfate was chosen as the bronchodilator. Before use, the drug solution was mixed well and inverted vertically, the patient wore a nose clip to prevent nasal aspiration, the subject was allowed to exhale deeply to the residual air volume level, and then a disposable mouthpiece was used to start a deep and slow inhalation through the mouth while the bronchodilator drug was sprayed out to allow the patient to inhale smoothly. At the end of the inhalation (total lung volume), the patient held his/her breath for 5-10 s. The procedure was repeated after 1 min until the pre-set inhalation dose was reached. For patients with a severe course of chronic obstructive pulmonary disease or poor cooperation, use an assistive device including a mist storage canister. A single dose of medication was sprayed into the canister, and then the subject was asked to complete a deep inhalation from the canister, hold his/her breath for 10 s, and then exhale again, with an interval of 1 min before the next spray was administered.

The operation of forceful spirometry was divided into 4 stages: 1) maximal inhalation after several even and calm breaths; 2) explosive exhalation with maximal force and maximum speed immediately after inhalation to the total lung volume level, 3) continuous exhalation up to the residual air level and 4) inhalation to the total lung volume. Synchronized volumetric time and flow volume curves were recorded, as well as Forced Vital Capacity (FVC), Forced Expiratory Volume in One Second (FEV1), and Forced Expiratory Volume in One Second as a percentage of Forced Vital Capacity (FEV1/FVC). Forced Expiratory Volume in One Second as a percentage of predicted value (FEV1%pred).

Exertion lung volume curves were considered acceptable if the following criteria were met: 1) no hesitation at the onset of expiration, adequate explosive force, steep and straight ascending branches of the expiratory curve, and a clear spike in maximum expiratory flow. 2) The evaluation of explosive force at the onset of expiration was performed using the extrapolation volume, which should be <5% of the expiratory lung volume or 0.150 L. 3) no coughing or pause within 1 second of the onset of the expiratory force, a smooth descent across the entire expiratory curve with no No sawtooth fluctuations, no premature inhalation, no leakage of air, etc. that would affect the results.


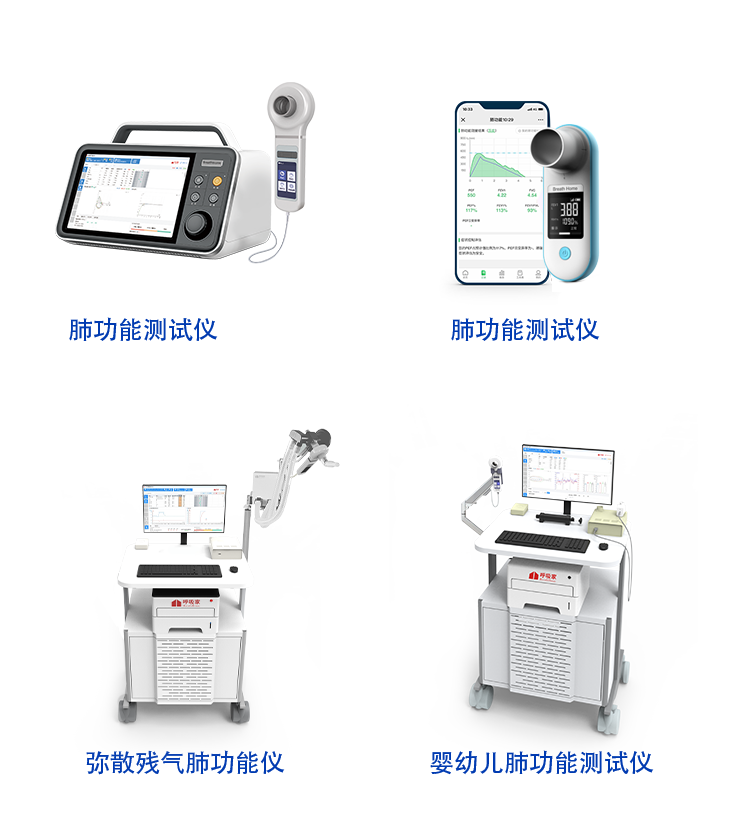


Figure1-1 Breathing Home BH-AX-MAPG Lung Function Monitor

（2）Maximal Inspiratory/Expiratory Pressure

Using the mouthpiece pressure acquisition system module of the spirometer, the pressure values (mmHg) in the Maximal Inspiratory Pressure (MIP) and Maximal Expiratory Pressure (MEP) states were recorded separately. For the MIP measurement, the subject was first required to continue the expiratory maneuver to the residual air volume position, and then to perform a maximal inspiration as hard as possible, holding the inspiratory state for 2 to 3 seconds. For the measurement of MEP, the subject should first inhale deeply to the total lung volume and then exhale to the best of their ability for a duration of 2 to 3 seconds.

(3) Diaphragm mobility and thickening scores

Diaphragm mobility and thickening score were used to determine the functional status of the diaphragm. The thickness of the diaphragm at the end of inspiration and at the end of expiration was recorded under calm breathing and deep breathing, respectively, and the diaphragm thickening score was calculated. The formula for calculating the diaphragm thickening score was: diaphragm thickness thickening rate = (maximum end-inspiratory thickness - calm end-expiratory thickness)/calm end-expiratory thickness×100%.

The patient was placed in the supine position with the head of the bed set at an angle between 0˚ and 30˚. Using a high-frequency line-array ultrasound probe, with the marker point facing upward, the probe was placed at the intersection of the patient's anterior axillary line and the 8th to 11th rib interspace of the mid-axillary line, and combined with the image to ensure that the probe was perpendicular to the skin of the chest wall. By adjusting the angle of the probe, the ultrasound beam was made perpendicular to the diaphragm. Based on the ultrasound image (e.g., Figure 2-2), three parallel echogenic structures can be observed, in which the upper and lower hyperechoic regions represent the pleura and peritoneum, respectively, while the middle hyperechoic region is the diaphragmatic layer that moves with respiration. The measured thickness of the diaphragm was the distance between the upper and lower hyperechoic regions (the thickness of the two hyperechoic regions was not included in the calculation). The thickness of the diaphragm was measured in B mode using a 5-12 MHz linear ultrasound probe, while the amplitude of diaphragmatic movement was measured in M mode by a 1-5 MHz convex ultrasound probe. Ultimately, the diaphragm thickening fraction was calculated from these data. The above measurements need to be repeated three times at the same position to ensure the accuracy of the data, and finally the average value was recorded.

For diaphragm mobility measurements, the patient remained in the supine position with the head of the bed set at a height between 0˚ and 30˚. At this point, a phased array probe or convex array probe was selected, with the probe marking point facing outward and downward and placed between the midclavicular and anterior axillary lines and at the junction of the costal arches, and the diaphragm image was obtained through the hepatic section. The probe should be aimed at the top of the diaphragm, which is shown on the right side as a hyperechoic linear structure overlying the liver surface and on the left side as a hyperechoic linear structure overlying the splenic surface. Mark the position of the diaphragm at end-expiration and end-inspiration, and the vertical distance between the two points is the degree of diaphragmatic movement.


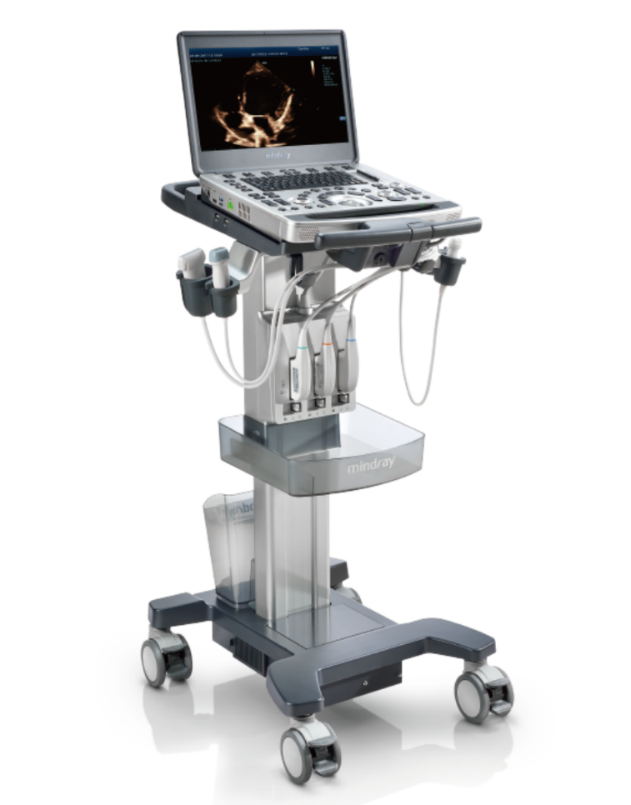


Figure 1-2 Myriad M9 Color Ultrasound Doppler Ultrasound


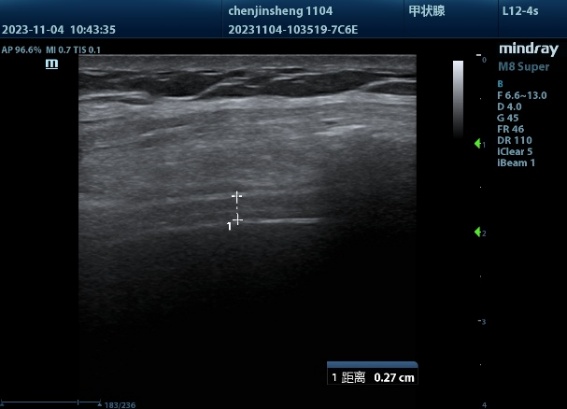

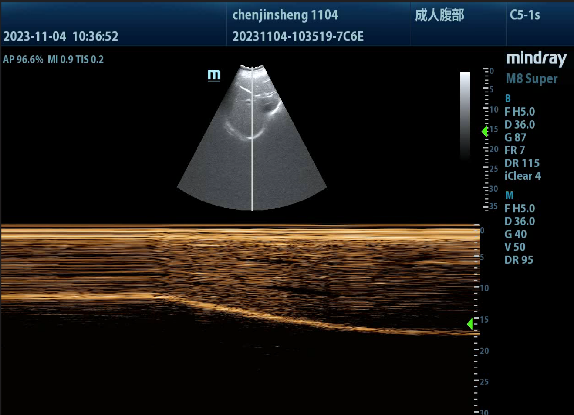


Figure 1-3 Ultrasound measurement of diaphragm thickness (left) and mobility (right)
